# Association between urinary manganese and blood pressure: Results from National Health and Nutrition Examination Survey (NHANES), 2011-2014

**DOI:** 10.1371/journal.pone.0188145

**Published:** 2017-11-15

**Authors:** Cynthia Wu, Jessica G. Woo, Nanhua Zhang

**Affiliations:** 1 Division of Biostatistics and Epidemiology, Cincinnati Children’s Hospital Medical Center, Cincinnati, OH, United States of America; 2 Krieger School of Arts and Sciences, Johns Hopkins University, Baltimore, Maryland, United States of America; The University of Tokyo, JAPAN

## Abstract

Manganese is a trace mineral required for metabolism, growth and tissue formation, and reproduction. It is mainly obtained through food and water, as well as through occupational exposure. This study used data from National Health and Nutrition Examination Survey, combining the 2011–12 and 2013–14 cycles. We conducted linear regression analyses on urinary manganese and blood pressure. Significant negative associations (p<0.01) between urinary manganese and both systolic and diastolic blood pressure existed after adjusting for age, sex, body mass index, race/ethnicity, and status of taking antihypertensive medication. These results indicate that urinary manganese may play some role in blood pressure and protecting against hypertension, a major risk factor for cardiovascular disease.

## Introduction

Manganese (Mn) is a trace mineral required at low concentrations for carbohydrate and lipid metabolism, growth and reproduction, and formation of tissues, and various enzymes—including ligases and hydrolases—are Mn-dependent [1]. Normal levels of manganese are 4–15 μg/L in blood and 1–8 μg/L in urine [2]. Excess exposure can result in manganism, which has many neurological symptoms similar to those of Parkinson’s disease—tremors, clumsy gait, speech disturbances, and muscle tension. Other potential health impacts include adverse reproductive, respiratory, and developmental effects. The primary pathway of exposure for the general population is through food and water. Workers in the mining and welding industries experience occupational exposure as well. Urinary excretion levels are most indicative of recent manganese exposures, as it can exit the body relatively quickly [2]. The liver removes excess manganese from the blood, and this manganese is then excreted into the bile and urine. The urinary excretion of manganese is not correlated with age or sex, but diabetics with liver disorders or those who were not treated with insulin tend to have significantly increased excretion of manganese [3].

Despite its label as a toxic heavy metal, manganese could potentially play a role in controlling blood pressure due to its anti-oxidative function [4]. The association between manganese and blood pressure has not been well-studied and remains somewhat controversial, but several previous studies have shown that the two may be negatively associated. Lee et al. [4] evaluated daily Mn intake in the Korean adult population and found that for men, it was significantly negatively correlated with systolic blood pressure. In another study, Mn-exposed milling workers had significantly higher incidence of diastolic hypotension compared to controls [5]. Mordukhovich et al. [6] reported that manganese was negatively associated—but not significantly—with both systolic and diastolic blood pressure.

In this study, we focused on urinary manganese and its relationship with blood pressure, using data from the National Health and Nutrition Examination Survey (2011–2014).

## Methods

### Study sample

The United States National Health and Nutrition Examination Survey (NHANES) is an annual cross-sectional survey conducted to assess the health of the population. Health interviews are conducted at the participants’ homes, and examinations and laboratory tests are also administered. The interview collects demographic, socioeconomic, and dietary data, and the exam and lab portions collect medical, dental, and physiological measurements. For this study, survey data from cycles 2011–2012 and 2013–2014 were used because previous years did not include measurements of urinary manganese. The study sample is representative of the national population.

### Urinary metals and blood pressure

Participants age six and over provided urine samples in the mobile examination center (MEC). These were analyzed using mass spectrometry, and the data were recorded into a database. All participants age eight and over had their blood pressure measured; examiners are certified for blood pressure measurement. Three consecutive blood pressure readings were obtained after the participant rested quietly in a seated position for 5 minutes and once the participant’s maximum inflation level was determined; a fourth attempt was made if a blood pressure measurement was interrupted or incomplete. An average value for both systolic and diastolic blood pressure was calculated and used, following the tutorial on NHANES website. Those excluded either did not fit the cuff, or had rashes, casts, edema, paralysis, tubes, open sores/wounds, withered arms, a-v shunts, or radical mastectomy.

### Covariates

Race/ethnicity was categorized as Mexican American, Other Hispanic, Non-Hispanic White, Non-Hispanic Black, Non-Hispanic Asian, and Other (including Multi-Racial). Missing values for antihypertensive medication were assumed to be “not taking medication”.

### Statistical analysis

The data were downloaded from the NHANES website and imported into R. The sample weights for the four years were calculated using the formula provided by NHANES. Other design features including stratification and clustering were also accounted for in all analyses. There were significant amounts of missingness in both blood pressure and urinary manganese (Table 1), with scattered missingness in the other covariates as well. The main reason for missingness was structural missingness (subjects not eligible for measurements due to age restrictions). Participants with missing outcomes or covariates were excluded from the analyses, with no subjects excluded due to missing data for age, sex, race/ethnicity or status of hypertension medication use. We compared those who were included in the analyses to those NHANES subjects who were excluded using contrast in the survey regression for continuous variables and the Rao-Scott Chi-square tests for discrete variables. Survey linear regression analyses between urinary manganese level and systolic and diastolic blood pressures were conducted. Model 1 reported the unadjusted analysis and model 2 adjusted for age, sex, race/ethnicity, body mass index (BMI), and status of taking antihypertensive medication. Pseudo *R*^2^ was calculated for both models based on deviances from the full model and the null model with intercept only [7]. All analyses were conducted using the “survey” package in R version 3.4.1.

**Table 1:** Characteristics of participants included in study population (N = 3853) and excluded participants (N = 15,452).

	Included in Analysis		Excluded from Analysis		
	N (%) with data	Mean ± SE	N (%) with data	Mean ± SE	p-value (included v. excluded)*
Overall N	3853		15,452		
Urinary manganese (μg/L)	3853	0.18 ± 0.02	1265	0.17±0.01	0.55
Age (years)	3853	37.34 ± 0.41	15,452	37.48±0.42	0.19
Body mass index (kg/m^2^)	3853	25.59 ± 0.07	13,249	25.20±0.07	<0.0001
Systolic blood pressure (mmHg)	3853	118.53 ± 0.15	10,306	118.62±0.19	0.49
Diastolic blood pressure (mmHg)	3853	65.90 ± 0.15	10,306	65.92±0.19	0.83
Status of antihypertensive medication	3853		15,452		<0.0001
Not taking or Missing	2732 (70.91%)		13,419 (86.84%)		
Taking	1121 (29.09%)		2033 (13.16%)		
Sex	3853		15,452		0.16
Female	1927 (50.01%)		7820 (50.61%)		
Male	1926 (49.99%)		7632 (49.39%)		
Race/ethnicity	3853		15,452		0.51
Mexican American	618 (16.04%)		2404 (15.56%)		
Other Hispanic	369 (9.58%)		1592 (10.30%)		
Non-Hispanic White	1294 (33.58%)		5138 (33.25%)		
Non-Hispanic Black	959 (24.89%)		3849 (24.91%)		
Non-Hispanic Asian	450 (11.68%)		1806 (11.69%)		
Other	163 (4.23%)		663 (4.29%)		

*p-values were based on F tests in contrasting the least square means in survey regression model for continuous variables, and based on the Rao-Scott Chi-square tests for discrete variables.

## Results

Out of the original sample size of 19305, 3853 participants were included in the study after excluding participants with missing values for the covariates and the variables of interest. The study population included 1927 females and 1926 males, and the mean age was approximately 37 years. Comparing characteristics of the participants included in the study and those excluded from the study, only BMI and status of taking antihypertensive medication resulted in significant differences (p<0.0001; Table 1). We attribute the difference between the groups’ BMI to the fact that younger children were more likely to be excluded from the study, since children under 8 did not have their blood pressure measured. Differences in medication may arise from the large number of “not taking medication” participants that were excluded, which also included missing values for this variable, again potentially due to the greater exclusion of younger children. However, approximately 29% of the study participants are “taking medication,” which is comparable to the 34% national prevalence of hypertension [8].

### Urinary manganese and blood pressure

The regression models of blood pressure on urinary manganese were shown in Table 2. Using the linear regression model and adjusting for age, sex, BMI, race/ethnicity, and status of taking antihypertensive medication, the regression coefficient of urinary manganese in the regression model of systolic blood pressure remained significantly negative (-1.313± 0.174, p<0.01), with a pseudo-*R*^2^ of 0.103.

**Table 2:** Linear regression analysis results for urinary manganese and blood pressure.

	Average Systolic Blood Pressure		Average Diastolic Blood Pressure	
	Estimate ± S.E. *	P-value	Estimate ± S.E.	P-value
Intercept	96.758 ± 1.700	< .0001	47.374 ± 1.478	< .0001
Urinary manganese (μg/L)	-1.313 ± 0.174	< .0001	-1.223 ± 0.200	< .0001
Age (years)	0.021 ± 0.015	0.1791	0.033 ± 0.013	0.0113
Body mass index (kg/m^2^)	0.758 ± 0.053	< .0001	0.641 ± 0.046	< .0001
Status of antihypertensive medication (Taking vs. Not taking)	-0.702 ± 0.797	0.3786	-0.019 ± 0.741	0.9795
Sex (Male vs. Female)	0.960 ± 0.751	0.2016	0.000 ± 0.640	0.9994
Race/ethnicity				
Mexican American	1.118 ± 0.998	0.2627	0.032 ± 0.841	0.9700
Other Hispanic	0.691 ± 1.120	0.5372	0.981 ± 0.968	0.3109
Non-Hispanic Black	-0.173 ± 0.796	0.8280	0.300 ± 0.674	0.6560
Non-Hispanic Asian	0.816 ± 1.042	0.4337	-0.377 ± 0.965	0.6960
Other	2.222 ± 1.883	0.2379	3.187 ± 1.429	0.0258
Non-Hispanic White	-	-	-	-
pseudo- *R*^2^	0.1028		0.1018	

*S.E.: Standard error.

Adjusting for age, sex, BMI, race/ethnicity, and status of taking antihypertensive medication, the association between urinary manganese and diastolic blood pressure remained significantly negative (-1.223± 0.200, p<0.01), with a pseudo-*R*^2^ of 0.102.

## Discussion

The 2011–2014 NHANES data from this study showed that higher urinary manganese levels were negatively and significantly associated with both systolic and diastolic blood pressure, even after adjusting for covariates. This potentially suggests that manganese protects against high blood pressure, a relationship that has been explored in previous studies but lacks strong, consistent evidence. Both Jiang and Zheng [5] and Lee et al. [4] reported negative correlations between blood pressure and manganese, although the latter studied daily intake rather than biomarkers. In another study, blood pressure was compared in male workers who were exposed to varying levels of manganese. The results showed that workers with the highest exposure had the lowest mean values of systolic blood pressure, but the same was not found with diastolic blood pressure [9]. Manganese also seems to inhibit myocardial contractions, dilate blood vessels, and induce hypotension [10].

This finding could have important implications since hypertension is a major cause of cardiovascular disease, which according to a 2017 report released by the American Heart Association [8], is still the leading cause of death in the United States. Not many studies have been conducted on manganese and its implications for treatment and prevention of cardiovascular outcomes. Oxidative stress seems to be a factor leading to hypertension because it increases vasoconstriction and disrupts endothelial function, which is important for vascular relaxation [11]. As previously stated, manganese may have anti-oxidative functions to combat this. Manganese can serve as a cofactor for superoxide dismutase (SOD) enzymes, which function to remove super oxides and other radical or reactive oxygen species. It can also form manganese-antioxidants (complexes of manganese) that function similar to SODs [12]. At this time, however, there is not enough research and evidence to know the extent of protection that manganese can provide against hypertension, and more future studies should focus on trace minerals like manganese.

There are several limitations to this study. First, urinary manganese may not be the most accurate measure of Mn exposure. Because of the limited number of studies available, a standard biomarker for measuring Mn exposure has not yet been established. Previous studies suggest that manganese in blood and urine can be used to detect higher-than-normal manganese levels in groups of people, but they are not as successful in reflecting individual Mn exposure [13,14]. This could be due partly to the fact that manganese is metabolized out of the body relatively quickly. However, in Lucchini et al. [15], results showed that individual levels of blood and urinary manganese were positively correlated with exposure after exposure had stopped. In another study comparing biomarkers of Mn exposure, toenail samples seemed to be best correlated with the cumulative Mn exposure index in the expected months after exposure, based on toenail growth rates [14]. However, blood and urinary manganese were not well correlated with toenail or air exposure over the course of the workday. Second, there were missing values for urinary Mn level, BMI, blood pressure, and antihypertensive medication status; with only 20% of the original data included in the final analysis, the results should be interpreted with caution. This leads to loss of efficiency due to exclusion of a large amount of data from the main analysis and diminishes the potential effect of medication on manganese levels. However, the study still has the advantage of retaining over 3800 subjects from a nationally representative sample. The structural missingness in Mn level and blood pressure could be viewed as random missingness and should not lead to biased estimate of the association between urinary Mn level and blood pressure. The third limitation of the study is the uncontrolled nature of Mn exposure, and therefore the relationship between urinary Mn level and blood pressure should not be interpreted as causal. This cross-sectional study using data from cycles 2011–2012 and 2013–2014 may be insufficient to study the effect of Mn level on blood pressure since it does not account for the duration of manganese exposure on the outcome. Although we have captured some of the key confounders–in particular age, BMI, and status of antihypertensive medication use–we cannot exclude the possibility that unobserved confounding remains.

## Conclusions

This study used two cycles of NHANES data from 2011 to 2014. Among the study population, there was a significant negative correlation between urinary manganese and systolic and diastolic blood pressure after adjusting for age, sex, BMI, race/ethnicity, and status of antihypertensive medication. Future research should focus on studying manganese and its relationship with blood pressure and cardiovascular outcomes, as well as establishing a standard biomarker of exposure for manganese.
